# Pre-TAVI aortic annulus sizing: comparison between manual and semi-automated new generation software measurements in operators with different experience

**DOI:** 10.1259/bjr.20220733

**Published:** 2023-07-25

**Authors:** Andrea Daniele Annoni, Maria Elisabetta Mancini, Eleonora Carlicchi, Marta Belmonte, Alberto Formenti, Saima Mushtaq, Francesca Marchetti, Francesco Cilia, Andrea Baggiano, Laura Fusini, Alice Bonomi, Marco Gennari, Piero Montorsi, Mauro Pepi, Gianluca Pontone

**Affiliations:** 1 Centro Cardiologico Monzino, IRCCS, Milan, Italy; 2 Centro Cardiologico Monzino, IRCCS, University of Milan, Milan, Italy; 3 ASST Grande Ospedale Metropolitano Niguarda, Milan, Italy; 4 Cardiovascular Center Aalst, OLV-Clinic, Aalst, Belgium; 5 Department of Clinical Sciences and Community Health, University of Milan, Milan, Italy

## Abstract

**Objectives:**

Aim of the study is to compare manual and semi-automatic measurements for aortic annulus assessment among different operators.

**Methods:**

Eighty patients who underwent TAVI were retrospectively enrolled. The measurements manually performed by an experienced reader for aortic annulus (minimum and maximum diameters, perimeter, area), annulus-to-coronary ostia distance and time needed for the whole evaluation, were collected. The same operator (observer 1) and two less experienced readers (observer 2 and 3, with >5 years and 1 year of experience, respectively) assessed the same measurements using a semi-automatic software. Differences between manual and semi-automatic measurements, reading time and suggested valves size derived by CT were compared.

**Results:**

Very good correlations were found between manual and software-aided measurements for aortic annulus area and perimeter in comparison with standard measurements for the three readers (ICC range 0.81–0.98). Good correlations were found for the distance with coronary ostia(0.75–0.79). The same area-derived prosthesis size for manual and semi-automatic measurements was selected in 96% of cases for observer 1; very good correlations were also found for observer 2 and 3 (ICC = 0.89 and 0.88, respectively). Using semi-automatic measurements, the mean time needed for CT images was significantly lower for observers 1 and 2 (1.50 and 1.72versus 3.14 min), respectively.

**Conclusions:**

Pre-TAVI CT using semi-automatic software allows accurate and reproducible measurements, reducing reconstruction time up to 50% and is reliable even for operators with different experience.

**Advances in knowledge:**

The use of semi-automatic dedicated software for CT in TAVI planning is reliable even for operators without long time experience and allows accurate and reproducible measurements improving pre-TAVI workflow.

## Introduction

### Background

Transcatheter aortic valve implantation (TAVI) is a widely established treatment strategy for patients with symptomatic, severe aortic stenosis (AS) who are at both intermediate and high risk for conventional surgical valve replacement.^1,2^ Nevertheless, not every patient considered at high-risk for surgery is suitable for TAVI. In addition to individual clinical risk scores, specific technical and anatomic criteria must be met because preprocedural annular sizing is performed on the basis of non-invasive imaging findings.^3^ Advances in non-invasive imaging have extensively supported growth of the field because the opportunity to have an accurate integration of advanced non-invasive imaging into patient selection, treatment planning, device selection, and device positioning. In this context, Computed Tomography (CT) has been demonstrated to be the gold standard noninvasive tool for annular sizing, evaluation of risk of annular injury, coronary occlusion, besides for peripheral access assessment.^4^ Nevertheless, pre-TAVI CT analysis requires accurate and time-consuming manual measurements that should be performed by experienced operator to ensure accuracy and reproducibility.

### Aortic annulus measurement techniques

Recent international guidelines^4,5^ report that Identification and positioning of the annular plane can be performed in different ways: manually, using standard multiplanar reformats, using a software-based facilitating workflow with manual identification of the basal hinge points by placing marker points with subsequent positioning of the plane by the software or by semi-automatically, by means of automated software-based anatomical segmentation. However in case of use of facilitated or semi-automated workflows the same guidelines strongly recommend that the accuracy of the generated annular plane must be verified by a trained observer and manual correction must be performed where required. The different tools used for annulus perimeter evaluation are reported to potentially lead to perimeter overestimation especially when manually placed segmentation points connected by straight lines without interpolation are used. Moreover, coronary ostial height measurements are strongly recommended to be performed using an electronic caliper tool in a perpendicular fashion to the annular plane from the annular plane to ensure reproducibility.

### Purpose

To the best of our knowledge, the use of semi-automatic software for pre-TAVI annulus and aortic root measurements in terms of accuracy, agreement and time in comparison with standard manual measurements has not been widely assessed. Moreover, most of the available published papers include pre-TAVI evaluations performed by experienced operators, not including the impact of different skills and differences regarding time-consuming measurements in the pre-procedural workflow. Thus aim of this study is to evaluate the accuracy, reproducibility and time saving of a semi-automated last generation software measurements performed by three operators with different experience, in comparison with manual measurements (considered as the reference standards) in patients that underwent TAVI procedure.

## Methods

### Study population

The study was approved by our institutional review board and informed consents for the anonymous publication of scientific data were obtained from all patients. 80 patients that underwent successful TAVI procedure with preprocedural CT exams were retrospectively selected for evaluation from our site’s internal database.

### CT scan protocol

CT examinations used for retrospective evaluation were conducted as stated in a study previously published by our group.^6^ CT scans were performed using a 256 slices wide volume coverage CT scanner (Revolution CT; GE Healthcare, Milwaukee, WI) without premedication with beta-blockers nor nitrates. A body mass index (BMI)-adapted protocol was used for tube current modulation. In all exams, retrospective ECG-gated with wide X-ray window of 500 ms in five distinct diastolic phases was used to assess the whole heart volume.

### CT images reconstruction and analysis

The original measurements as pre-procedure planning were manually performed for each patient using a dedicated workstation (Advantage Workstation VolumeShare 4.6, GE Healthcare with specific VesselIQ Xpress software) by an experienced reader (Reader 1, 15 years of experience in cardiovascular CT acquisition and reconstruction) for aortic annulus dimensions (minimum and maximum diameters, perimeter and area) and coronary ostia height. Measurements and the time needed for the whole evaluation, were collected. As part of the site’s standard practice, during clinical work up, measurements are usually performed at least twice. However, only the final results decided by the reading physician are recorded in the patient’s record. In this retrospective study, the same measurements were performed by three readers, including reader 1, and two less experienced readers (reader 2 and reader 3, with >5 years and 1 year of experience, respectively) using a dedicated software (TAVI Analysis, GE Healthcare). A wash out period ranging between 3 weeks minimum and 10 months occurred prior to reader 1 participating in the review of TAVI exams with the semi-automated TAVI software. Reader 2 and reader 3 did not participate in the manual reading for the 80 patients in their pre-procedural planning. Readers were blinded to each other’s results and to the original clinical measurements. Differences between manual and semi-automatic measurements and the reading time were compared. The suggested CT area-derived valve sizes were compared with the implanted prosthesis size (Manual Method).

Aortic annulus measurements were performed in an orthogonal plane on the center line of the aorta as described by previous studies from our group^6^ and recommended by the Society of Cardiovascular Computed Tomography (SCCT) expert consensus document.^4^ To orientate the aortic annulus to the annular plane, the reader used the double oblique tools prior to making measurements. The measurements were performed using the manual length measurement tool for the diameter measurements. For quantification of annular area and perimeter a polygon measurement tool was used with manually placed segmentation points automatically connected by straight lines. Coronary ostial heights were measured in a perpendicular fashion to the annular plane using a caliper tool from the annular plane to the lower edge of the coronary artery ostium. The measurements generated by this method were referred as standard reference (based on previous procedural success). The CT-derived valve size obtained were compared with the implanted valve size.

### TAVI software analysis method

**Figure 1.**
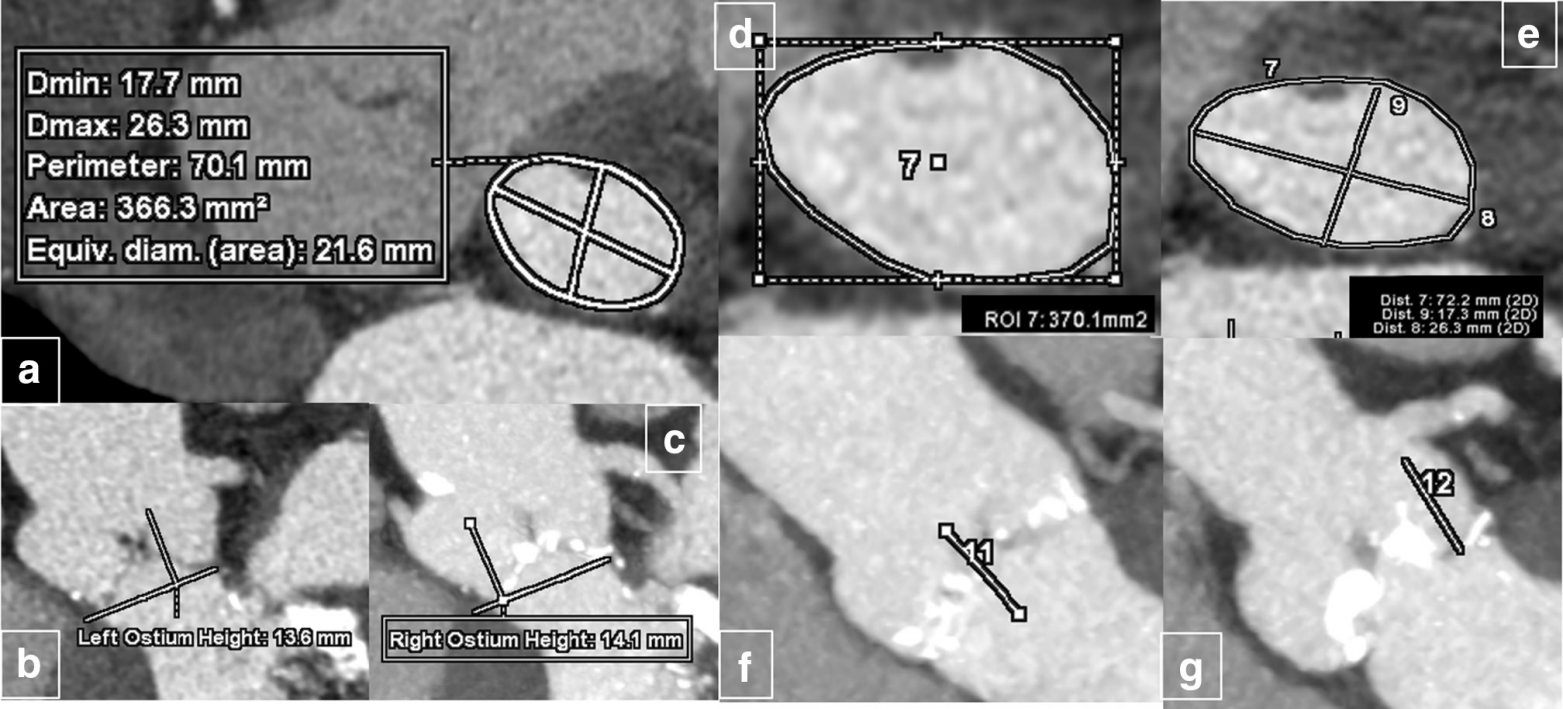
Semi-automatic software-aided aortic annulus measurements (left side) including annulus area, perimeter and diameters (**a**) and the distance from coronary ostia (**b,c**) in comparison with standard measurements (right side-panels) showing annulus area (**d**), annulus diameters and perimeter (**e**) and the distance from the coronary arteries ostia (**f,g**). The measurements were performed in the same patient with good measures overlapping.

For TAVI software analysis method, each reader launched each dataset into the TAVI analysis protocol. Following the software, the aortic annulus plane selection was set by the reader depositing a point on each coronary cusp hinge point. The software uses the three points to set the annulus plane and provide a pre-contour of the annulus. Accuracy of the generated annular plane was then confirmed by the operator analyzing the imaging that performed manual corrections if required. After validating the contour, the software displays the identified measurements of minimum and maximum diameter, perimeter and area of the annulus plane. Coronaries ostial height measurements were performed by placing a point at the lower edge of the coronary artery ostia: the software automatically displays the measures from the annulus plane previously assessed. The measurements and the time to generate the whole measurements were recorded. Moreover for each patient, the CT-derived valve size was compared to the actual size of the implanted valve prosthesis (resulting from a multidisciplinary discussion including CT measurements, ecocardiography findings and operator’s preference regarding available prosthesis models). Figure 1 shows measurements performed with different methods.

### Statistical analysis

Statistical analysis was performed with the SPSS v. 25.0 software (SPSS Inc, Chicago, IL). Continuous variables were expressed as mean ± SD. Discrete variables were expressed as absolute numbers and percentages. Cohen’s κ with its 95% confidence interval (CI) was used to quantify interobserver reliability for nominal variables. The agreement between operators and between software and manual measurements method was measured by the intraclass correlation coefficient (ICC) and the coefficient of variation (CV). ICC values less than 0.5 were considered as poor reliability, values between 0.5 and 0.75 were considered as moderate-good reliability, values between 0.75 and 0.9 indicated good reliability, and values > 0.90 indicate excellent reliability.^7^ The Bland-Altman diagram was applied to compare measurements of aortic annulus dimensions distance from the annulus to the coronary ostia from all three readers performed with dedicate software with the reference standard measurements.

## Results

### Study population characteristics

We selected 80 patients (44 males and 36 females) from our internal database. Mean age was 81.61 ± 5.23 years and mean body mass index (BMI) 25.45 ± 4.61 Kg/m^2^. Mean heart rate during CT acquisition was 71.31 ± 10.25 beats per minute with seven patients with atrial fibrillation. All the selected patients underwent successful TAVI procedure. Study population characteristics are listed in Table 1.

**Table 1.** Patients population characteristics

Age (years)	81.61 ± 5.23
Male / Female.	44/36
BMI (KG/m^2^)	25.45 ± 4.61
HR (beats/min)	71.31 ± 10.25
LVEF (%)	50.0 ± 7.22
Aortic valve gradient (max mmHg)	74.51 ± 7.10
Atrial fibrillation	7/80

BMI, body mass index; HR, heart rate; LVEF, left ventricle ejection fraction.

### Intra- and interobserver variability and correlation

**Figure 2.**
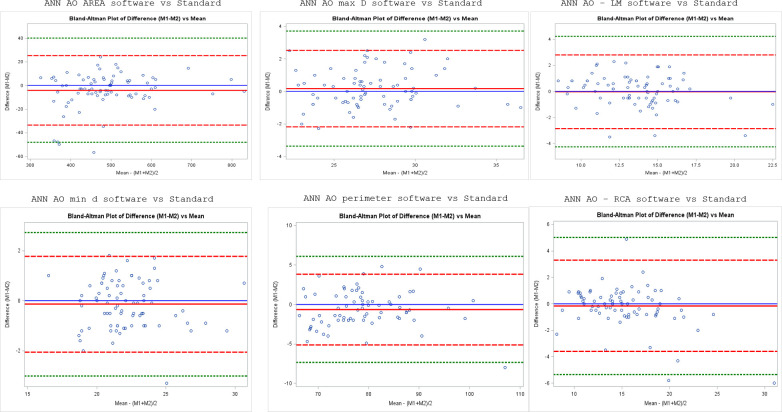
Bland-Altman diagrams for the average aortic annular measurements from all three readers in correlation with the reference standard measurements (ANN_AO_AREA: aortic annulus area; ANN_AO_d: aortic annulus minimum diameter; ANN_AO_D1; aortic annulus maximum diameter; ANN_AO_PER: aortic annulus perimeter; ann___LM: distance between aortic annulus and left main; ann___RCA: distance between aortic annulus and right coronary artery)

Very good intraclass coefficient was found between manual and TAVI software-aided measurements for aortic annulus area and perimeter among all the observers (0.91 and 0.86, respectively). Very good correlations were found between manual and software-aided measurements performed by observer 1. Good correlations were found regarding annulus diameters (0.75–0.76) and distance from annulus plane and coronary ostia (0.75–0.79). Very good intraclass coefficients values were also found regarding the suggested valve size (0.89) (Table 2). Table 3 reports the intraclass correlations between standard and software-aided measurements for each observer. The best performance was observed in measurements performed by observers 1 and 2. Moreover, observers 1 and 2 showed similar high correlations coefficient about measurements regarding aortic annulus area, perimeter and suggested valve. Very good correlations were found for the other measurements. Observer 3 measurements showed lower correlations coefficients even if maintaining very good values regarding area and suggested valve size (ICC 0.89 and 0.88, respectively). Very good correlations were found between the CT-derived valve size measurements and the size of the implanted valve prosthesis: on average the CT area-derived prosthesis size from the TAVI software measurement by all three readers showed ICC of 0.89 in comparison with the actual implanted valve. Very good correlations were also found for each reader (ICC = 0.94, 0.89, and 0.88 for reader 1, 2,and 3, respectively). Figure 2 shows Bland-Altman diagrams for the average aortic annular measurements from all three readers in correlation with the reference standard measurements.

**Table 2.** Intraclass correlation between manual and TAVI software-aided measurements among all the observers

variable	Mean_variable	Measurement_error	Variation coefficient	INTRACLASS COEFFICIENT (ICC)
AREA (mm^2^)	486.68	31.28	0.064	0.91
Min diameter (mm)	22.03	1.23	0.056	0.76
Max Diameter (mm)	27.28	1.47	0.054	0.75
Perimeter (mm)	78.58	3.07	0.039	0.86
ann___LM (mm)	13.33	1.27	0.095	0.75
ann___RCA (mm)	15.27	1.73	0.11	0.79
suggested_valve	26.17	0.67	0.025	0.90

AREA, aortic annulus area;Max Diameter, aortic annulus maximum diameter; Min diameter, aortic annulus minimum diameter; Perimeter, aortic annulus perimeter; ann___LM, distance between aortic annulus and left main; ann___RCA, distance between aortic annulus and right coronary artery; suggested_valve, suggested valve size.

**Table 3.** Intraclass correlation between manual and TAVI software aided measurements for each observer

Standard *vs* SFTW OBS 1					
**variable**	**Mean_variable**	**Measurement_error**	**Variation coefficient**	**INTRACLASS COEFFICIENT (ICC**)	
AREA (mm^2^)	488.50	10.70	0.021	0.98	
Min diameter (mm)	22.14	0.67	0.030	0.92	
Max Diameter (mm)	27.45	0.83	0.030	0.91	
Perimeter (mm)	78.96	1.63	0.020	0.95	
ann___LM (mm)	13.60	0.99	0.073	0.85	
ann___RCA (mm)	15.33	1.21	0.079	0.90	
suggested_valve	26.07	0.47	0.018	0.94	
**Standard** ***vs*** **SFTW** **OBS 2**					
**variable**	**Mean_variable**	**Measurement_error**	**Repeatibility**	**C_V**	**ICC**
AREA (mm^2^)	488.20	18.69	51.79	0.038	0.96
Min diameter (mm)	22.11	0.85	2.38	0.038	0.87
Max Diameter (mm)	27.36	1.05	2.92	0.038	0.86
Perimeter (mm)	78.73	1.99	5.52	0.025	0.93
ann___LM (mm)	13.38	1.04	2.88	0.077	0.84
ann___RCA (mm)	15.26	1.33	3.69	0.087	0.88
suggested_valve	26.11	0.67	1.85	0.025	0.89
**Standard** ***vs*** **SFTW** **OBS 3**					
**variable**	**Mean_variable**	**Measurement_error**	**Repeatibility**	**C_V**	**ICC**
AREA (mm^2^)	489.05	33.92	93.97	0.06	0.89
Min diameter (mm)	22.14	1.37	3.80	0.06	0.72
Max Diameter (mm)	27.27	1.62	4.49	0.05	0.70
Perimeter (mm)	78.88	3.59	9.95	0.04	0.81
ann___LM (mm)	13.44	1.45	4.03	0.10	0.72
ann___RCA (mm)	15.46	1.90	5.27	0.12	0.78
suggested_valve	26.13	0.71	1.97	0.02	0.88

AREA, aortic annulus area; C_V, Coefficient of variation; Max Diameter, aortic annulus maximum diameter; Min diameter, aortic annulus minimum diameter; Perimeter, aortic annulus perimeter; ann___LM, distance between aortic annulus and left main; ann___RCA, distance between aortic annulus and right coronary artery; suggested_valve, suggested valve size.

### Reconstruction time

The mean time needed for the whole evaluation was significantly lower for reader one in comparison with the time needed for measurements performed with the standard tool (3.14 ± 0.49 min *vs* 1.50 ± 0.39 min). Mean time for reader 2 and reader 3 were 1.72 ± 0.41 and 2.92 ± 0.54 min, respectively. To notice that the mean time recorded for the less experienced operator was slightly lower than the mean time recorded with standard tool by the more experienced reader (2.92 ± 0.54 *vs* 3.14 ± 0.54 min) (Table 4).

**Table 4.** Time needed for measurements with standard tool and semi-automated software

Time				
	**op**	**Measures n**.	**Mean time (min**)	**Standard deviation**
**SFTW**	**1**	**80**	1.50	0.39
**SFTW**	**2**	**80**	1.72	0.41
**SFTW**	**3**	**80**	2.92	0.54
**STANDARD**	**1**	**80**	3.14	0.49

Measures n, measures number; Op, operator; SFTW, software; min, minutes.

## Discussion

Our study represents one of the few actual comparisons between manual and semi-automated pre-TAVI CT measurements using this specific software in operators with different experience skills. The main finding of the study is that semi-automated software allows accurate pre TAVI CT measurements in comparison with the standard manual measurements tools even in operators without extensive expertise. Moreover, the semiautomated software showed improvements in the workflow reducing the time needed for the whole CT measurements (quite halving the time needed for more experienced operators).

### Pre-TAVI CT

It has been widely demonstrated that although not a sole source of information in the case of TAVI pre-procedure planning, CT is typically used to help determine aortic annulus size, to guide selection of appropriate valve, provide dimensions of the entire aorta and give guidance for deployment of the device to improve the procedure outcome and for risk assessment such as prosthesis migration, annular rupture, and coronary ostia occlusion.^8,9^ This requires accurate measurements from CT images performed by experienced operators regardless of what tools used, and review of such measurements by the dedicated clinical care team (the structural heart team). In this scenario, technological improvements regarding new CT scanner have contributed to improve diagnostic performance of this imaging modality that remains the most accurate and powerful tool for accurate assessment of aortic annulus, aortic root, lef ventricular outfow tract, coronary-ostia height and vascualr accesses, in order to potentially prevent severe complications.^9^ This process applies to the standard manual measurements as well as semi-automatic measurements outputted from TAVI. Therefore, the final valve type and size is determined by this comprehensive clinical review through a rigorous process by an experienced heart team for each patient. Available studies regarding pre-TAVI measurements reproducibility are mainly focusing on experienced readers using manual tools. In the last years, further studies assessed the use of specific software for analyzing the CT images for TAVI planning similarly focusing on experienced readers even in comparison with different imaging modalities.^10–14^

### Readers experience comparison evaluation

Previously published guidelines by the European Society of Cardiology and the American College of Cardiology have reported a minimum requirements in terms of number of cases interpreted and learning hours to guarantee a good accuracy for coronary CT reconstruction and report^15,16^ Whereas for pre-TAVI CT assessment, only few studies suggest the need for a learning curve for inexperienced readers.^17^ Our study results improve data regarding the reliability of using a semi-automated software for CT images analysis in TAVI planning by demonstrating good correlation and reproducibility for measurements even in less experienced readers. Moreover in comparison with previous studies, we not only used a new generation software not widely evaluated before but also included operators with significant experience difference in cardiovascular CT reconstruction including operators with wide expertise range (1 to 15 years) Considering our results concerning aortic annulus area, per convention and consideration of the published literature,^18^ the Limits of agreement (LoA) was chosen to be ±1.96 σ which translates to the expectation that 95% of the results should lie within the LoA. Inspection of the plots shows that 93.8% of the results are within the LoA for average of 3 reader’s measurement. Therefore, of the total of the 240 results, 224/240 (93.3%) are within the LoA without a systematic bias between the TAVI-aided measurements and reference standard with the mean close to zero. Moreover, the average aortic annular perimeter from all three readers had a very high positive correlation to the reference standard measurement (ICC 0.86). Since our institution primarily uses annulus area to suggest valves, our results showed very good correlations between the CT-derived valve size and the implanted prosthesis. Reader 1’s measurements have a high degree of success in suggesting the right valve sizes (ICC 0.94) while reader 2’s and reader 3’s measurements showed lower correlation even if with ICC values indicating very good reliability.^19^,^20^ This could not be unexpected given their level of experience in this clinical area. Nevertheless, it is also important to note that in clinical practice, the CT measurements are not used in isolation for determining the recommended valve size. This is commonly done within the Heart Team as part of the full case review taking into consideration all the clinical information about the patient, all imaging results and the overall shape of the annulus. Moreover even considering the reader’s measurements, the 3/80 patients in which implanted valve sizes differed from what the standard care method would suggest illustrates precisely importance of the role of the comprehensive real-world clinical practice employed by the heart team in patient care played. Therefore, it is conceivable that final sizes for implantation would have been determined through the same comprehensive review process, as in the standard manual measurement process, generating the same clinical outcome.

### Impact on pre-procedural workflow

Additionally, our results suggest that the use of a semi-automated TAVI software can lead to faster measurements process and workflow. Additionally, considering the time estimates for performing the CT analysis, semi-automatic measurements time recorded with the use of TAVI software was significantly lower for readers 1 and 2 and slightly lower for reader 3. As expected, the reader experience appears to have a direct correlation to the overall processing time. Although the third reader’s time was not as significantly reduced as compared to readers 1 and 2 our results show that there is still an efficiency gain when compared to the existing standard of care workflow.

### Study limitations

Some limitations to our study must be acknowledged. First, this is a single-center study. Second, we compared semi-automatic software measurements of three observers with different expertise levels while the manual measurements used for comparison were only performed by one experienced reader. Third, we didn’t include patients with bicuspid aortic valves in whom the annulus assessment could be more challenging. Fourth, our study was focused on measurements for aortic annulus and aortic root, excluding the aortic measurements regarding the peripheral accesses.

## Conclusions

In conclusion, the use of semi-automatic software can improve pre-TAVI CT analysis workflow allowing accurate and reproducible measurements. Moreover, the use of a dedicated software can reduce the time needed for reconstructions up to 50% in comparison with the standard reformat-tool and appears reliable also for operators with different expertise.
